# The Impact of an In-service Educational Program on Nurses' Knowledge and Attitudes Regarding Pain Management in an Ethiopian University Hospital

**DOI:** 10.3389/fpubh.2018.00229

**Published:** 2018-08-20

**Authors:** Gugsa N. Germossa, Ingeborg Strømseng Sjetne, Ragnhild Hellesø

**Affiliations:** ^1^School of Nursing and Midwifery, Jimma University Institute of Health Sciences, Jimma University, Jimma, Ethiopia; ^2^Department of Nursing Sciences, Faculty of Medicine, Institute of Health and Society, University of Oslo, Oslo, Norway; ^3^Division of Health Services, Norwegian Institute of Public Health, Oslo, Norway

**Keywords:** nurses, pain management, educational programs, knowledge, attitudes, Ethiopia

## Abstract

**Background:** Although pain control for hospitalized patients is a central issue for all health care providers, nurses' knowledge, and attitudes are the major barriers. Educational program is a strategy to improve nurses' knowledge and attitudes on pain management. However, there is paucity of information on how in-service education program influences nurses' knowledge and attitudes score for pain management in Ethiopia. The objective of this study was therefore, to investigate the influence of an in-service educational program on nurses' knowledge and attitudes regarding pain management in an Ethiopian university hospital.

**Methods:** A quasi-experimental study was conducted between 1 October and 15 November 2016. Totally 111 nurses working at Jimma University Medical Center participated in the study. We provided 2 consecutive days of intensive pain management education with a follow-up training session after 1 month. Knowledge and Attitudes Survey Regarding Pain (KASRP) was used as a tool for measuring the impact of educational program. Data were analyzed using the Wilcoxon signed-rank test, and results were considered significant at *p* < 0.05.

**Result:** Of the 111 nurses, who participated in the study, 39.5% were female, 46.8% had a baccalaureate degree, and 67.6% had worked in nursing for 6–10 years. The mean age of respondents was 26.9 (*SD* ± 5.6) years. On average, participants answered 41.4% of the survey items correctly before the intervention and 63.0% after the intervention. The mean rank score of nurses' knowledge and attitudes regarding pain significantly improved following participation in the educational program (*Z* = −9.08, *p* < 0.001).

**Conclusion:** The educational program improved nurses' scores for pain management knowledge and attitudes. This may lead to more effective pain management by nurses.

## Introduction

Pain, the oldest clinical problem remains undertreated among hospitalized patients (1–4). The experience of pain from pathological conditions, medical procedures, trauma, and childbirth makes pain management very important in hospital settings (4). Alleviation of pain is an important nursing goal embodied in the profession's philosophy (5, 6). Nurses are responsible for regular pain assessment, medication administration, and monitoring of the patient's responses. These responsibilities require an understanding of the nature of pain in relation to a patient's clinical condition (7).

Although pain control for hospitalized patients is a central issue for all health care providers, major barriers are presented by nurses' inadequate knowledge, negative attitudes, insufficient assessment skills, reluctance to act as the patient's advocate (8, 9), and misconceptions (7, 9). Inadequate pain management reflects inadequate knowledge on the part of nurses. adequate pain knowledge helps nurses to underpin their practices of pain assessment, medication administration, and monitoring (10). Nurses' attitudes and beliefs may influence patient care (11). Evidence show that nurses having adequate knowledge and good attitude of pain may lead to more effective pain management (12). Nurses who believe in the importance of patient pain relief implement more pain management activities (13), and nurses with positive attitudes will have the intention and motivation to provide care for a patient with pain (11).

In Ethiopian university hospitals, the prevalence of inadequately managed pain is as high as 80.1% (14). This is higher than levels reported for other countries: 30–80% for Hong Kong (15), 45% for Italy (16), and 79.7% for Jordan (17). System failure, providers' misconceptions, inappropriate beliefs, opiophobia, and lack of knowledge (8, 14) are among the numerous factors contributing to inadequate pain management in general (8) and in Ethiopia in particular (14).

Previous studies have shown that in-service educational programs improved nurses' knowledge and attitudes regarding pain management (18–20). Nevertheless, levels of education and standards of nursing practice may vary within and between countries. For example, almost half of nurses in Ethiopia practicing in public hospital lack adequate knowledge of pain (21).

Although nurses in Ethiopian hospitals may have different levels of training backgrounds, they hold the same position and have the same bedside responsibilities (22). The level of nurses' knowledge and attitudes regarding pain is directly linked to their training during pre-service education. However, curriculum reviews indicate a lack of emphasis on pain during pre-service educational programs (22), and 50% of recently graduated nurses in Ethiopia lack adequate knowledge of pain management (21). Nurses can also acquire further knowledge of pain through work experience, in-service education, and interaction with colleagues (23). We have not identified studies investigating how in-service education program influences nurses' knowledge and attitudes score for pain management in Ethiopian university hospitals.

Thus, the aim of this study was to investigate how an in-service educational program influenced nurses' knowledge and attitudes regarding pain management in an Ethiopian university hospital.

## Materials and methods

### Study design and setting

A quasi-experimental study design with a pre-test-post-test approach was used in Jimma University Medical Center (JUMC) from 1 October to 15 November 2016. JUMC is the only teaching and referral hospital in the southwest of Ethiopia. It serves a population of 15 million people. The hospital has 600 inpatient beds in different wards (medical, surgical, gynecology, maternity, pediatric, neonatal, ICU, psychiatric, and ophthalmological). The present study was carried out in the medical, surgical, maternity, and gynecology wards. We recruited all 165 staff nurses involved in the provision of bedside nursing care in these wards. Their knowledge and attitudes regarding pain management were measured before intervention (pre-test) and again after intervention (post-test).

### Intervention

An in-service education program was arranged in rounds of training. In each round, 30–40 nurses participated. The educational program was delivered in three ways: 2 consecutive days of intensive in-person sessions (16 h of face-to-face training), provision of reading materials to facilitate take-home reading assignments (self-learning), and refresher training 4 weeks later (8 h). The trainers were two doctors (a pediatrician and an internal medicine specialist) and two nurses (the principal investigator and a palliative care Ph.D. fellow). All the trainers hold “training of trainer” certificates on pain management from the Ethiopian Federal Ministry of Health (FMOH). The training was delivered by means of interactive lectures, group discussions, practical exercises, case scenarios, and take-home reading assignments.

The content of the educational program was developed based on the FMOH (Federal Ministry of Health) pain management guideline (24), standard nursing textbooks (25–28), WHO guidelines (29, 30), and relevant literature (2, 7, 31). It was tailored to the four domains of pain management competency (7): the multidimensional nature of pain, pain assessment and measurement, management of pain, and clinical conditions. The educational sessions covered the following areas:

- Introduction (definition, mechanisms)
- Classifications (nociceptive, neuropathic breakthrough, emergency, and incident pain)
- Pain assessment using a numerical rating scale and practical exercises
- Historical myths, cultural barriers
- Subjective nature of pain, and professional misconceptions
- General pain management and treatment (use of opioids, the WHO analgesics ladder, opioid side effects and toxicity)
- Addiction and dependency
- Pain treatment in children, HIV/AIDS, pregnancy and childbirth, and old age
- Non-pharmacological approaches to pain
- Patient education and counseling

The training manual and presentation materials were given in hard copy and soft copies on compact disks and memory sticks, which contained selected research articles and reference manuals. The principal investigator was accessible by phone around the clock to clarify issues related to pain management.

### Data collection procedure

Initial contact with nurse participants was made through head nurses on the relevant wards. A code for each nurse was generated from the ward name lists, and each nurse was told to remember his or her code. The researcher then distributed an enveloped document containing the survey tool and the consent form. Nurses were asked to sign the consent form to show their willingness to participate in the study. The survey took 30–45 min, and the completed questionnaires were collected and stored in the head nurse's office.

### Data collection instrument

We used the revised version of the Knowledge and Attitudes Survey Regarding Pain (KASRP) developed by Ferrell et al. (32). To our knowledge, it is the only available tool for measuring knowledge and attitudes of health professionals in relation to pain. It consists of 41 items: 22 true/false questions, 15 multiple-choice questions, and two cases with two responses each. It has been used all over the world since 1987 and revised over the years to reflect changes in pain management practice. The content of the tool is derived from current standards of pain management from bodies including the American Pain Society and the World Health Organization and from the comprehensive national Cancer Network Pain Guidelines (32). At the time of development, it had a test-retest reliability of *r* > 0.80 and an internal consistency reliability of α > 0.70 (32). Each correctly answered item is assigned a score of 1; a score of 0 is assigned otherwise. A respondent's total score can range from 0 (the lowest possible score) to 41 (the highest possible score). The author of the survey questionnaire warns researchers against differentiating items as measuring either knowledge or attitudes. Since many items measure both knowledge and attitudes (for example, the item that measures the incidence of addiction), the developers recommend analyzing the responses in terms of the percentages, complete scores, and individual items answered independently by each respondent. We also collected information on nurse characteristics (work unit, gender, educational level, prior in-service training, and work experience). The questionnaire was administered in English, the main language of instruction in the Ethiopian education system.

### Data analysis

Data were checked for completeness and accuracy before being entered to statistical software package SPSS version 20.1 (IBM SPSS Statistics for Windows, Armonk, NY). Descriptive statistics were used to describe the sample characteristics and the responses to each item. Because the post-test-pre-test score differences violated assumptions for normality, we performed non-parametric tests. The Wilcoxon signed-rank test was used to analyze differences in the rank of KASRP scores before and after the educational program. McNamara's test was used to analyze differences in the proportions of correct answers for each item before and after the program. The Mann–Whitney U test was used to analyze differences in the mean rank of pre-test and post-test KASRP scores and sample characteristics (gender, educational level, and prior in-service training). The Kruskal–Wallis H test was performed to analyze differences in the mean rank of pre-test and post-test KASRP scores and the nurses' work unit. A *p*-value of < 0.05 was considered significant for all statistical tests.

### Ethical consideration

The study has been notified to the Data Protection Officer for Research, NSD—Norwegian Center for Research Data (project number 48349). Before the commencement of data collection, ethical approval was sought from the institutional review board of the College of Health Science of JUMC, and administrative permission was obtained from JUMC. The head nurse was instructed not to keep any records of participants who did not return a completed questionnaire. During data collection, written informed consent was obtained from each participant. Upon completion of the educational program, each participant was awarded a certificate.

## Results

### Sample characteristics

Before the educational program, 165 survey questionnaires were distributed; 124 were returned, of which 120 were complete. During the post-test survey, 120 questionnaires were distributed; 114 were returned, of which 111 were complete. The mean age of the respondents was 26.9 years (*SD* ± 5.6). Table 1 shows the distribution of participants by gender, prior in-service training on pain, educational level, work experience, and work unit.

**Table 1:** Demographic and professional characteristics (*N* = 111).

**Characteristic**	***n* (%)**
Gender	
Female	44 (39.6)
Male	66 (59.5)
Missing	1 (0.9)
Prior in-service training on pain	
Yes	12 (10.8)
No	99 (89.2)
Educational level	
Diploma	58 (52.3)
BSc degree	52 (46.8)
Missing	1 (0.9)
Work experience	
< 1 year	13 (11.7)
2–5 years	75 (67.6)
6–10 years	13 (11.7)
More than 10 years	6 (5.4)
Missing	4 (3.6)
Working unit	
Gynecology ward	11 (9.9)
Medical ward	28 (25.2)
Surgical ward	50 (45.0)
Maternity ward	22 (19.8)

### Nurses' knowledge and attitudes regarding pain management

Except for two respondents whose scores remained the same, all respondents increased their performance on KASRP at post-test. On average, participants answered 41.4% of the survey items correctly before the intervention and 63.0% after the intervention. Our findings showed that the mean score on KASRP increased after intervention from 17 (*SD* ± 4.0) to 25.8 (*SD* ± 7.2) (Table 2). Except for two items those measures the effectiveness of non-steroidal anti-inflammatory agent in painful bone metastases and probability of respiratory depression when taking stable doses of opioids, the proportion of correct answers for each item in the survey after the intervention significantly generally increased. (Supplementary Table 1).

**Table 2:** Proportion of correct answer and mean pre-test and post-test KASRP^a^ scores.

**Test**	**Proportion of correct answer**	**Mean (95% CI)**	**Minimum**	**Maximum**
Post-test score	63.0	25.8 (24.4–27.1)	13	38
Pre-test score	41.4	17.0 (16.3–17.8)	10	36
Difference	21.6	8.8 (7.6–10.0)		
Test statistic		*t* = 14.1		
*p*-value		<0.001		

a *Knowledge and Attitudes Survey Regarding Pain*.

The mean rank score of nurses' knowledge and attitudes regarding pain significantly improved following participation in the educational program (*Z* = −9.08, *p* < 0.001) with a large effect size (*r* = 0.61). However, the sample characteristics show some variation in levels of knowledge and development of attitudes at post-test (Table 3). The result showed that nurses who had not received prior in-service education on pain had a higher pre-test score (17.1) compared with nurses who had received such education (16.1). Nurses who had not received prior in-service education on pain had the largest difference in post-test-pre-test scores as well.

**Table 3:** Distribution of mean KASRP^a^ score by sample characteristic.

**Characteristic**	**Pre-test score**	**Post-test score**	**Post-test-pre-test score difference**	***P*-value**
Gender				0.12
Male	16.6	24.7	8.1	
Female	17.6	27.6	10.1	
Prior in-service education on pain				0.03
Yes	16.1	21.1	5.0	
No	17.1	26.3	9.3	
Level of education				0.40
Diploma	16.4	24.8	8.4	
Degree	17.6	27.0	9.4	
Length of service				1.00
< 1 year	15.4	23.5	8.2	
2–5 years	17.4	26.4	9.0	
6–10 years	17.3	26.2	9.0	
More than 10 years	14.7	25.3	10.7	
Working unit				< 0.05^b^
Maternity ward	14.6	18.9	4.4	
Gynecology ward	18.1	26.4	8.3	
Medical ward	17.0	27.3	10.4	
Surgical ward	17.8	27.8	10.0	

a *Knowledge and Attitudes Survey Regarding Pain*.

b *The mean difference is significant at P < 0.05 and the difference is between maternity ward and surgical ward, and maternity ward and medical ward*.

As shown in Table 3, there was no statistically significant difference observed in KASRP scores improvement according to the nurses' education level. Greater improvements on KASRP scores at post-test was observed among female nurses compared with male nurses. Regarding working unit, nurses in surgical, medical, and gynecology wards scored higher than those in maternity wards (65.1, 61.8, and 59.2, respectively, compared to 26.3; *p* < 0.05).

## Discussion

Our findings show a significant improvement in scores for knowledge and attitudes regarding pain management following participation in the educational program. It provides an important information about the beneficial impact of an educational program on nurses' knowledge and attitudes regarding pain management. After completion of the educational program, 98.2% all participants increased their score on KASRP. This indicates improved knowledge and attitudes regarding pain for the cohort. On average, the proportion correctly answered survey items increased by 52% following the intervention.

The level of nurses' understanding and their attitudes regarding pain management may be linked to the adequacy of care they provide for patient in pain. Knowledge, the capacity to behave and perform actions with full understanding is acquired through learning, practice, and interaction with environments. To be useful, knowledge must not only be acquired but also remembered or retained (23). Attitude refers to a mental state of readiness and reaction (positive or negative) to a phenomenon. Knowledge and attitudes are learned and shaped by cumulative experiences in life (11). Nurses can therefore modify their preexisting knowledge and attitudes toward pain by participating in an educational program designed to promote better knowledge and improved attitudes. Pre-testing of participants' knowledge and attitudes prior to an educational program provides a way to detect and identify deficiencies in knowledge and attitudes. Post-testing following completion of an educational program is important for establishing how much knowledge has been retained and the extent to which attitudes have changed.

Our findings indicate that nurses' knowledge before the educational program was limited, which is consistent with other similar studies (18, 20, 33). This may be because nurses receive inadequate preparation for pain management in their pre-service education. As evidenced by the KASRP test scores, the current study demonstrates that the educational program was effective in improving nurses' knowledge and changing their attitudes toward pain management. However, the level of improvement significantly varied by item, prior in-service education on pain, and work unit. The average number of correct answers on the survey significantly improved after the educational program, which is consistent with previous studies (18–20). An unexpected finding was that 12 nurses who had not received prior in-service education on pain had higher pre-test and post-test scores compared with nurses who had received prior in-service education. We lack information that could be used to explore the reason for this result. For example, when the other nurses received the in-service education. This could be a subject for future studies.

Although the proportion of items answered correctly increased from 41.4 to 63% following the educational program, this falls far below expectations and indicates the need for further continuous education tailored to the needs of nurses. In-person, face-to-face learning sessions integrated with self-learning are required, and additional strategies such as web-based instruction and distance learning should be customized to current levels of understanding to reach many nurses with minimal cost. The importance of continuing education has also been reported in other studies (19).

In a complex intervention with multiple components (including classroom teaching by pain experts, use of printouts and electronic copies to guide self-learning, and refresher sessions), it is difficult to attribute the contribution of each specific component to the final results (34). Within the scope of this study, we could determine only the impact of the whole intervention against baseline scores for knowledge and attitudes. The educational program in the present study upgraded nurses' understanding of pain management. This might help them to carry out patient pain assessments, aligning analgesics to pain severity levels and monitoring patient responses to treatment more confidently. Because pre-service preparation for pain management might not be adequate (7, 24), designing and implementing short in-service educational programs may be an effective strategy for addressing gaps in nursing care relating to pain control.

Other studies have reported that educational programs can improve nurses' knowledge and attitudes regarding pain (12, 20, 33) and that improved knowledge may lead to better and effective pain management (12). However, changes in nurses' knowledge and attitudes alone may not suffice. Improving the clinical practice of hospitalized pain management will require sustained effort, together with the formation of multidisciplinary teams and the implementation of system-wide changes that monitor and keep health care professionals to pay attention and to control pain in accordance with the recommended guidelines. To facilitate sustainable changes in clinical practice, it is necessary to incorporate pain management into nurses' workflow while providing continuous in-service refresher education and information. Other important facilitators that may enhance nursing actions include rounding and integration of the pre-service curriculum.

Many factors may function as limitations of the current study: the use of a non-randomized design without control, the possibility of guessing when answering survey items (as with any test that includes true/false and multiple-choice questions), and the possibility that nurses may have gained additional knowledge and improved their attitudes in interaction with medical professionals. Initially, we planned to employ a quasi-experimental design with control. However, our freedom of movement was restricted by a period of public unrest followed by a declaration of a state of emergency. This made it impossible to travel between the intervention and control sites, and our plans to use a control group had to be abandoned. A larger sample would also have strengthened the study.

A further limitation relates to the fact that most of the diploma nurses participating in the study were also attending other evening or weekend upgrading programs at different levels, and this could have impacted the post-test results. This may also be the reason for the absence of statistical differences between the test performances of degree-educated and diploma-educated nurses. It should also be borne in mind that the program in the present study involved the distribution of resources designed to enhance self-learning. Given all these factors, a simple pre-test-post-test design with two measurement points over a 6-week period is an inadequate basis for causal inferences. Further randomized, multicenter studies are necessary before attributing improvements in knowledge and attitudes to the effects of an educational program and before such a program can be recommended for other professions. However, the present findings indicate the potential benefits of a mixed in-service educational program (incorporating both face-to-face sessions and self-learning) for enhancing nurses' knowledge and attitudes regarding pain management for hospitalized patients, despite the resource-intensive nature of such programs. Comparison with less expensive educational programs, such as web-based and distance learning programs, will be important in identifying the most effective approach.

The current study provides empirical evidence that an educational program significantly improved nurses' knowledge and attitudes score regarding pain management in an Ethiopian university hospital. It could have been better to correlate change in nurses' knowledge and attitude with change in pain management practices that might leads to improvements in patients' experience of pain, thus further study is required to investigate the impact of an-in-service educational program on patients' experiences with pain management.

## Author contributions

GG contributed conception, design of the study, and wrote the first draft of the manuscript. IS and RH revised the proposal and wrote sections of the manuscript. All the authors contributed to statistical analysis, fieldwork designing, and manuscript revision, read and approved the submitted version. All authors confirm that the manuscript is our own original work.

### Conflict of interest statement

The authors declare that the research was conducted in the absence of any commercial or financial relationships that could be construed as a potential conflict of interest.

## Abbreviations

ETH: Ethiopia
FMOH: Ethiopian Federal Ministry of Health
JUMC: Jimma University Medical Center
WHO: World Health Organization
KASRP: Knowledge and Attitudes Survey Regarding Pain
NORAD: Norwegian Agency for Development Cooperation
NORHED: The Norwegian Program for Capacity Development in Higher Education and Research for Development.
