# Supplementary material for: The Impact of an In-service Educational Program on Nurses' Knowledge and Attitudes Regarding Pain Management in an Ethiopian University Hospital
Source: Front Public Health. 2018 Aug 20;6:229. doi: 10.3389/fpubh.2018.00229 (PMC6110152; doi:10.3389/fpubh.2018.00229)
Supplement: Supplementary file 1 [file Table_1.pdf]

Supplementary material- Proportion of correct answers on KARP items before and after intervention

Items: True/false statements(correct answer)	Percent correct answer (N=111)		
	Pre-test	Post-test	McNemar's test
	n (%)	n (%)	
Vital signs are always reliable indicators of the intensity of a patient's pain. (False)	39 (35.1)	59 (53.3)	***
Because their nervous system is underdeveloped, children under two years of age have decreased pain sensitivity and limited memory of painful experiences. (False)	68 (61.3)	90 (81.1)	***
Patients who can be distracted from pain usually do not have severe pain. (False)	63 (51.6)	89 (80.2)	***
Patients may sleep in spite of severe pain. (True)	35 (31.5)	64 (57.7)	***
Aspirin and other non-steroidal anti-inflammatory agents are NOT effective analgesics for painful bone metastases. (False)	55 (49.5)	62 (55.5)	*
Respiratory depression rarely occurs in patients who have been receiving stable doses of opioids over a period of months. (True)	78 (70.3)	87 (78.4)	*
Combining analgesics that work by different mechanisms (e.g., combining an NSAID with an opioid) may result in better pain control with fewer side effects than using a single analgesic agent. (True)	76 (68.5)	92 (82.9)	***
The usual duration of analgesia of 1–2 mg morphine IV is 4–5 hours. (False)	39 (35.1)	59 (53.3)	***
Opioids should not be used in patients with a history of substance abuse. (F)	32 (28.8)	70 (63.1)	***
Elderly patients cannot tolerate opioids for pain relief. (False)	42 (37.8)	68 (61.3)	***
Patients should be encouraged to endure as much pain as possible before using an opioid. (False)	29 (26.1)	58 (52.3)	***
Children less than 11 years old cannot reliably report pain so clinicians should rely solely on the parent's assessment of the child's pain intensity. (False)	39 (35.1)	76 (68.5)	***
Patients' spiritual beliefs may lead them to think pain and suffering are necessary. (True)	72 (64.9)	89 (80.2)	***
After an initial dose of opioid analgesic is given, subsequent doses should be adjusted in accordance with the individual patient's response. (True)	83 (74.8)	98 (88.3)	***
Giving patients sterile water by injection (placebo) is a useful test to determine if the pain is real. (False)	22 (19.8)	61 (55.0)	***

Vicodin (hydrocodone 5 mg + acetaminophen 300 mg) PO is approximately equal to 5–10 mg of morphine PO. (True)	50 (45.0)	72 (64.9)	***
If the source of the patient's pain is unknown, opioids should not be used during the pain evaluation period, as this could mask the ability to correctly diagnose the cause of pain. (False)	20 (18.0)	59 (53.2)	***
Anticonvulsant drugs such as gabapentin (Neurontin) produce optimal pain relief after a single dose. (False)	27 (24.3)	50 (45.0)	***
Benzodiazepines are not effective pain relievers and are rarely recommended as part of an analgesic regiment. (True)	67 (60.4)	79 (71.2)	
Narcotic/opioid addiction is defined as a chronic neurobiologic disease, characterized by behaviors that include one or more of the following: impaired control over drug use, compulsive use, continued use despite harm, and craving. (True)	77 (69.4)	96 (86.5)	***
The term 'equianalgesia' means approximately equal analgesia and is used when referring to the doses of various analgesics that provide approximately the same amount of pain relief. (True)	79 (71.2)	92 (82.9)	**
Sedation assessment is recommended during opioid pain management because excessive sedation precedes opioid-induced respiratory depression. (True)	78 (70.3)	92 (82.9)	***

*P Value > 0.05, ** PValue < 0.01, ***P Value ≤ 0.001

The recommended route of administration of opioid analgesics for patients with persistent cancer-related pain is (oral).	15 (13.5)	54 (48.6)	***
The recommended route administration of opioid analgesics for patients with brief, severe pain of sudden onset such as trauma or post-operative pain is (intravenous).	71 (64.0)	88 (79.2)	***
Which of the following analgesic medications is considered the drug of choice for the treatment of prolonged moderate to severe pain for cancer patients? (morphine)	84 (75.7)	99 (89.2)	***
A 30 mg dose of oral morphine is approximately equivalent to (morphine 10 mg IV).	27 (24.3)	58 (52.3)	***
Analgesics for post-operative pain should initially be given (around the clock on fixed schedule).	62 (55.9)	80 (72.1)	***
A patient with persistent cancer pain has been receiving daily opioid analgesics for 2 months. Yesterday the patient was receiving morphine 200 mg/hour intravenously. Today he has been receiving 250 mg/hour intravenously. The likelihood of the patient developing clinically significant respiratory depression in the absence of new co morbidity is (less than 1%).	21 (18.9)	48 (43.2)	***
The most likely reason a patient with pain would request increased doses of pain medication is (patient experienced increased pain).	42 (37.8)	69 (62.2)	***
Which of the following is useful for treatment of cancer pain? (All: Ibuprofen, gabapentin, and hydromorphine.)	44 (39.6)	65 (58.6)	***
The most accurate judge of the intensity of the patient's pain is (the patient).	39 (35.1)	67 (60.4)	***
Which of the following describes the best approach for cultural considerations in caring for patients in pain? (Patient should be individually assessed to determine cultural influences.)	45 (40.5)	70 (63.1)	***
How likely is it that those patients who develop pain already have an alcohol and/or drug abuse problem? (5–15%)	44 (39.6)	59 (53.2)	**
The time to peak effect for morphine given IV is (15 minutes).	76 (68.5)	93 (83.8)	***
The time to peak effect for morphine given orally is (1–2 hours).	32 (28.8)	48 (23.2)	
Following abrupt discontinuation of an opioid, physical dependence is manifested by the following: (sweating, yawning, diarrhea, and agitation).	17 (15.3)	37 (33.3)	***
Which statement is true regarding opioid-induced respiratory depression? (Obstructive sleep apnea is an important risk factor.)	39 (35.1)	55 (45.5)	**
Patient A: Andrew is 25 years old and this is his first day following abdominal surgery. As you enter his room, he smiles at you and continues talking and joking with his visitor. Your assessment reveals the following information: BP = 120/80; HR = 80; R = 18; on a scale of	16 (14.4)	72 (64.9)	***

0 to 10 (0 = no pain/discomfort, 10 = worst pain/discomfort), he rates his pain as 8. On the patient's record you must mark his pain on the scale below. Circle the number that represents your assessment of Andrew's pain. (8)			
Your assessment, above, is made two hours after he received morphine 2 mg IV. Half-hourly pain ratings following the injection ranged from 6 to 8, and he had no clinically significant respiratory depression, sedation or other untoward side effects. He has identified 2/10 as an acceptable level of pain relief. His physician's order for analgesia is "morphine IV 1–3 mg q1h PRN pain relief." Check action you will take at this time. (Administer morphine 3 mg IV now.)	10 (9.0)	40 (36.0)	***
Patient B: Robert is 25 years old and this is his first day following abdominal surgery. He is lying quietly in bed and grimaces as he turns in bed. Your assessment reveals the following information BP = 120/80; HR = 80; R = 18; on a scale of 0 to 10 (0 = no pain/discomfort, 10 = worst pain/discomfort), he rates his pain as 8. On the patient's record you must mark his pain on the scale below. Circle the number that represents your assessment of Robert's pain. (8)	21 (18.9)	70 (63.1)	***
Your assessment, above, is made two hours after he received morphine 2 mg IV. Half-hourly pain ratings following the injection ranged from 6 to 8, and he had no clinically significant respiratory depression, sedation, or other untoward side effects. He has identified 2/10 as an acceptable level of pain relief. His physician's order for analgesia is "morphine IV 1–3 mg q1h PRN pain relief." Check action you will take at this time. (Administer morphine 3 mg IV now.)	8 (7.2)	26 (23.1)	***
